# CEP-1347 Dually Targets MDM4 and PKC to Activate p53 and Inhibit the Growth of Uveal Melanoma Cells

**DOI:** 10.3390/cancers16010118

**Published:** 2023-12-25

**Authors:** Keita Togashi, Shuhei Suzuki, Yuta Mitobe, Yurika Nakagawa-Saito, Asuka Sugai, Senri Takenouchi, Masahiko Sugimoto, Chifumi Kitanaka, Masashi Okada

**Affiliations:** 1 Department of Molecular Cancer Science, School of Medicine, Yamagata University, 2-2-2 Iida-nishi, Yamagata 990-9585, Japan; 2 Department of Ophthalmology and Visual Sciences, School of Medicine, Yamagata University, 2-2-2 Iida-nishi, Yamagata 990-9585, Japan; 3 Department of Clinical Oncology, School of Medicine, Yamagata University, 2-2-2 Iida-nishi, Yamagata 990-9585, Japan; 4 Department of Neurosurgery, School of Medicine, Yamagata University, 2-2-2 Iida-nishi, Yamagata 990-9585, Japan; 5 Research Institute for Promotion of Medical Sciences, Faculty of Medicine, Yamagata University, 2-2-2 Iida-nishi, Yamagata 990-9585, Japan

**Keywords:** intraocular malignancy, MDMX, KT7515, choroid, ciliary body, iris

## Abstract

**Simple Summary:**

Uveal melanoma (UM) is a rare cancer that forms in the eye. Once the disease becomes metastatic, which occurs in approximately half of the cases, the prognosis is highly dismal with there being no standard treatment, underscoring the dire need to develop novel systemic therapies for UM. In this study, we discovered that two molecular features characteristic of UM, MDM4 overexpression and increased protein kinase C (PKC) activity associated with infrequent p53 mutations and frequent GNAQ/GNA11 mutations, respectively, work together to suppress p53 to confer growth advantage on UM cells. Furthermore, we show that a small-molecule kinase inhibitor CEP-1347 targets MDM4 and PKC at the same time to effectively activate p53 and inhibit the growth of UM cells at clinically feasible concentrations. Thus, CEP-1347 is a unique, dual inhibitor of MDM4 and PKC in UM cells and may become a therapeutic option for refractory UM.

**Abstract:**

Uveal melanoma (UM) is among the most common primary intraocular neoplasms in adults, with limited therapeutic options for advanced/metastatic disease. Since UM is characterized by infrequent p53 mutation coupled with the overexpression of MDM4, a major negative regulator of p53, we aimed to investigate in this study the effects on UM cells of CEP-1347, a novel MDM4 inhibitor with a known safety profile in humans. We also examined the impact of CEP-1347 on the protein kinase C (PKC) pathway, known to play a pivotal role in UM cell growth. High-grade UM cell lines were used to analyze the effects of genetic and pharmacological inhibition of MDM4 and PKC, respectively, as well as those of CEP-1347 treatment, on p53 expression and cell viability. The results showed that, at its clinically relevant concentrations, CEP-1347 reduced not only MDM4 expression but also PKC activity, activated the p53 pathway, and effectively inhibited the growth of UM cells. Importantly, whereas inhibition of either MDM4 expression or PKC activity alone failed to efficiently activate p53 and inhibit cell growth, inhibition of both resulted in effective activation of p53 and inhibition of cell growth. These data suggest that there exists a hitherto unrecognized interaction between MDM4 and PKC to inactivate the p53-dependent growth control in UM cells. CEP-1347, which dually targets MDM4 and PKC, could therefore be a promising therapeutic candidate in the treatment of UM.

## 1. Introduction

Although uveal melanoma (UM) is a rare subtype of melanoma, it is one of the most common primary intraocular malignancies in adults and is genetically distinct from cutaneous malignant melanoma [1,2,3]. Eye-preserving treatments, such as radiation therapy, heavy ion therapy, and local excision, are performed for early-stage UM. However, advanced cases do not respond to local therapy, and due to the lack of effective chemotherapy, large lesions, lesions with vitreous seeds, and lesions with optic nerve involvement require enucleation of the eye [4]. Moreover, approximately 50% of UM patients develop distant metastases in the liver and other organs via the bloodstream, resulting in a poor prognosis [3,5,6]. Metastatic UM is often resistant to chemotherapy [7], and immune checkpoint inhibitors, such as PD-1 antibodies, have limited effects [8,9,10]. Due to the lack of effective drugs, novel therapies for UM are needed to improve the prognosis of advanced cases, such as metastatic UM, and locally recurrent cases.

*TP53*, a tumor suppressor gene that encodes the p53 protein, is frequently mutated in human cancers. However, unlike other human cancers, the p53 mutation is relatively rare in UM [6,11]. Instead, the function of wild-type p53 in UM is often inactivated by negative regulators of p53, such as murine double minute 2 (MDM2) and MDM4 (MDMX). Previous studies demonstrated that MDM2 and MDM4 were highly expressed in UM and contributed to cell proliferation [11,12]. Based on these findings, small molecules that induce p53 reactivation may be effective against UM. These agents, such as Nutlin3a, target the interaction of p53 with MDM2 or the MDM2/MDM4 complex; however, since MDM2 is also involved in the suppression of p53 in normal cells, these small molecules also induce a p53 response in normal cells and exert adverse effects, such as myelosuppression [13,14,15]. Therefore, the MDM2 inhibitors developed to date may not be optimal, and agents that activate the p53 pathway with fewer adverse effects are needed.

We previously reported that CEP-1347, a drug developed for the treatment of Parkinson’s disease that has a well-characterized safety profile, acted as a novel MDM4 inhibitor for p53 stabilization and activation by suppressing MDM4 expression in retinoblastoma, glioblastoma, and meningioma cells [16,17,18,19]. However, the efficacy of CEP-1347 in the treatment of UM still remains unexplored. In the present study, therefore, we examined the effects of CEP-1347 on UM cells.

## 2. Materials and Methods

### 2.1. Reagents and Antibodies

CEP-1347 was purchased from TOCRIS Bioscience (Bristol, UK). Gö6976, Crystal Violet (C.I. 42555), and Trypan Blue solution (0.4%) were obtained from Merck KGaA (Darmstadt, Germany). Propidium iodide and Hoechst33342 were supplied by Thermo Fisher Scientific (Waltham, MA, USA). An antibody against MDM4 (A700-000-T) was purchased from BETHYL (FORTIS LIFE SCIENCES, Waltham, MA, USA). An antibody against MDM2 (AF1244) was purchased from R&D Systems (Minneapolis, MN, USA). A p53 antibody (sc-126) was from Santa Cruz Biotechnology, Inc. (Santa Cruz, CA, USA). Antibodies against GAPDH (#5174), p21 Waf1/Cip1 (cyclin-dependent kinase inhibitor 1A, CDKN1A) (#2947), Myristoylated alanine-rich protein kinase C (PKC) substrate (MARCKS) (#5607), Phospho-MARCKS (Ser152/156) (#2741), Phospho-PKC (pan) (βII Ser660) (#9371), Phospho-PKCα/β II (Thr638/641) (#9375), Phospho-PKCδ (Thr505) (#9374), and Phospho-PKCθ (Thr538) (#9377) were from Cell Signaling Technology, Inc. (Beverly, MA, USA).

### 2.2. Cell Culture

Human UM cell lines, Mel202 and 92.1, were obtained from the European Collection of Authenticated Cell Cultures (Salisbury, UK). Normal human fetal lung fibroblasts (IMR90) were from the American Type Culture Collection (Manassas, VA, USA). Primary human UM cells (UM-Y01) were established from surgical tissues obtained after informed consent from a UM patient (a 75-year-old male, spindle-B cell type UM [Grade 1] without distant metastasis) in accordance with a protocol approved by Yamagata University School of Medicine. Tumors dissected into small fragments with scissors were, after being washed with PBS, incubated in TrypLE Express (Thermo Fisher Scientific) at 37 °C for 30 min. The enzyme-treated fragments were filtered through a 70 μm strainer (Corning, AZ, USA), and dissociated cells were cultured in DMEM/F12 thereafter as UM-Y01. Mel202 and 92.1 cells were maintained in RPMI-1640 medium. IMR90 cells were maintained in DMEM, and cells with a low passage number (<8) were used. Culture media were supplemented with fetal bovine serum (10%), penicillin-streptomycin mixed solution (100 U/mL, and 100 μg/mL).

### 2.3. Cell Viability Assays

The WST-8 assay was conducted as previously described using Cell Counting Kit-8 (DOJINDO LABORATORIES, Kumamoto, Japan) [20]. The trypan blue dye exclusion assay was performed as previously described using Trypan Blue solution [18]. The propidium iodide incorporation assay was performed as previously described [20].

### 2.4. Immunoblot Analysis

An immunoblot analysis was conducted as previously described [17]. Cells were lysed with RIPA buffer (10 mM Tris/HCl [pH 7.4], 0.1% sodium dodecyl sulfate [SDS], 0.1% sodium deoxycholate, 1% Nonidet P-40, 150 mM NaCl, 1 mM EDTA, 1.5 mM sodium orthovanadate, 10 mM sodium fluoride, 10 mM sodium pyrophosphate, and protease inhibitor cocktail set III [FUJIFILM Wako Chemicals, Osaka, Japan]). Protein samples whose concentrations were measured using the BCA kit (Pierce Biotechnology, Inc., Rockford, IL, USA) were separated by SDS-PAGE, transferred to PVDF membranes, and reacted with primary antibodies, then detected using Immobilon Western Chemiluminescent HRP Substrate (Merck Millipore, Burlington, MA, USA) and detected by a ChemiDoc Touch device (Bio-Rad Laboratories, Inc., Hercules, CA, USA). Original immunoblot images are shown in Figure S1.

### 2.5. Transfection of siRNA

Using Lipofectamine RNAiMAX (Thermo Fisher Scientific) in accordance with the manufacturer’s instructions, Stealth RNAi siRNA Negative Control Med GC Duplex #2 (200 pmol per 6 cm dish) and siRNAs against human p53 (#2: HSS186390 and #3: HSS186391; 120–160 pmol per 6 cm dish) or human MDM4 (#1: HSS106417 and #2: HSS106418; 160–200 pmol per 6 cm dish) (Thermo Fisher Scientific) were transfected.

## 3. Results

### 3.1. CEP-1347 Suppresses Cell Viability in UM Cells

**Figure 1:**
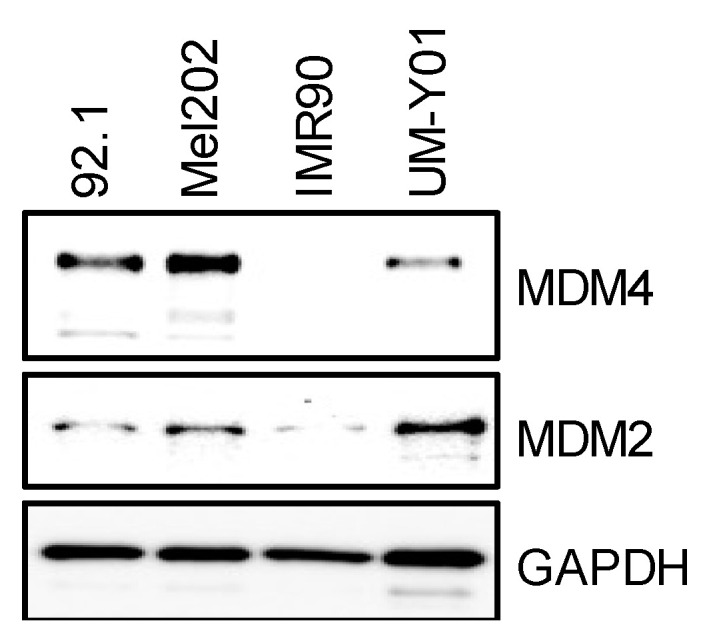
MDM4 is highly expressed in uveal melanoma cells. The expression status of murine double minute 4 (MDM4) and MDM2 in uveal melanoma cells (92.1, Mel202, and UM-Y01) and human normal fibroblasts (IMR90). The indicated cells, cultured without any drug treatment, were subjected to an immunoblot analysis of the indicated proteins.

**Figure 2:**
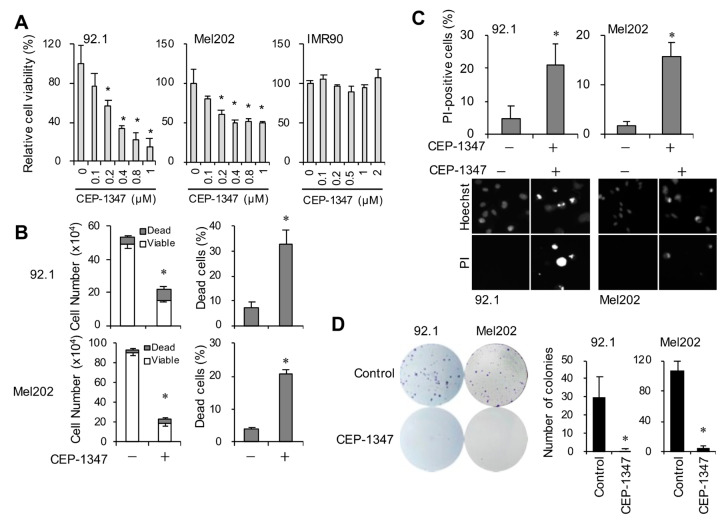
CEP-1347 induces growth inhibition and cell death in uveal melanoma (UM) cells. (**A**) The relative cell viability of human UM cell lines (92.1 and Mel202) and human normal fibroblasts (IMR90) treated with CEP-1347 for 3 days was determined by the WST-8 assay. (**B**,**C**) UM cell lines were treated with CEP-1347 (0.5 μM, 3 days). The numbers of viable and dead cells (left panels) as well as the percentage of dead cells (right panels) were determined by the trypan blue dye exclusion assay (**B**), or alternatively, the percentage of dead cells was determined by the propidium iodide incorporation assay (**C**, top panels). Representative images are shown (**C**, bottom panels). (**D**) Colony formation assay. Cells treated with CEP-1347 (0.5 μM) for 3 days and then cultured in its absence for another 10 days were stained with crystal violet. The values in a representative experiment are demonstrated as the means ± SD of triplicate samples. * *p* < 0.05 vs. treated without CEP-1347 (i.e., at 0 μM) by the Student’s *t*-test. Similar results were obtained from more than two separate biological replicates.

First, we investigated whether CEP-1347 suppressed, similarly to retinoblastoma and glioblastoma cells, the viability of UM cells using high-grade UM cell lines (92.1 and Mel202: epithelioid melanoma, Grade 3) [21,22] and a cell line established from a patient with low-grade UM (UM-Y01: spindle cell melanoma, Grade 1). We initially compared MDM4 expression levels in these UM cell lines and in IMR90, normal human fibroblasts. MDM2 and MDM4 expression levels were higher in the UM cell lines than in IMR90, and the expression of MDM4, but not MDM2, increased in a grade-dependent manner (Figure 1). We therefore investigated first whether CEP-1347 inhibited the viability of the high-grade UM cells, which expressed its target molecule MDM4 at high levels, using the WST assay. While CEP-1347 did not suppress cell viability in IMR90 at a concentration of approximately 2 μM, it significantly inhibited the viability of high-grade UM 92.1 and Mel202 cells at ≥200 nM, well within the clinically relevant concentration range (Figure 2A) [23,24]. To establish whether the decrease in metabolic activity observed in the WST assay reflected a reduction in the number of viable cells and an increase in cell death, we performed the dye exclusion test. The results obtained showed that CEP-1347 reduced the number of viable cells and induced cell death in UM cells (Figure 2B,C). We subsequently performed the colony formation assay to investigate whether CEP-1347 inhibited the long-term clonogenic viability of UM cells and demonstrated that it significantly and effectively reduced the number of colonies (Figure 2D). Collectively, these results demonstrated the potent inhibitory effects of CEP-1347 on the viability of UM cells expressing wild-type p53 and high levels of MDM4.

### 3.2. CEP-1347 Reduces MDM4 Expression and Activates p53 in UM Cells

**Figure 3:**
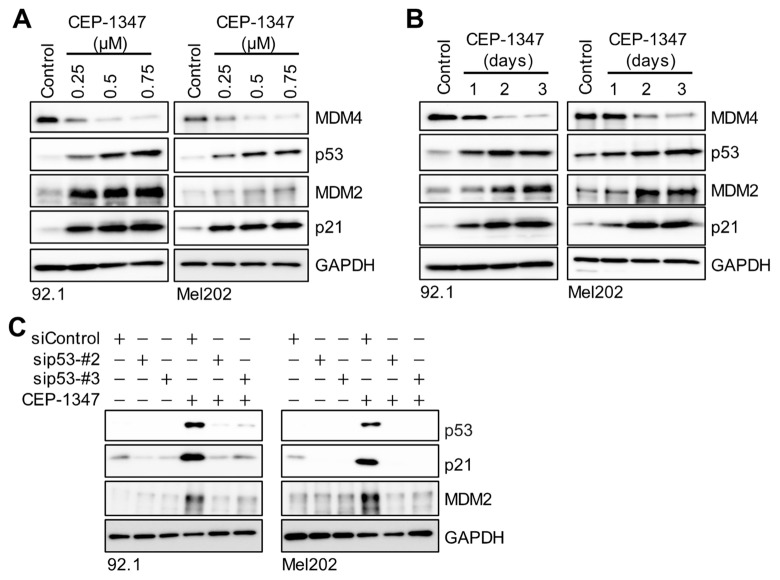
CEP-1347 decreases MDM4 expression in uveal melanoma cells to activate the p53 pathway. (**A**,**B**) Immunoblot analysis of 92.1 and Mel202 cells treated as indicated with CEP-1347. (**C**) Immunoblot analysis of cells transiently transfected with an siRNA against p53 (#2 or #3) or a control siRNA (siControl). One day after transfection, cells were treated with or without CEP-1347 (0.5 μM, 3 days). Similar results were reproduced in two independent biological replicates.

We next investigated whether CEP-1347 inhibited the viability of UM cells by activating the p53 pathway. To achieve this, we initially assessed the expression levels of p53-related factors in CEP-1347-treated UM cells using immunoblotting. We demonstrated that the treatment with CEP-1347 down-regulated the expression of MDM4 and up-regulated that of p53 in 92.1 and Mel202 cells in concentration- (Figure 3A) and time- (Figure 3B) dependent manners. Furthermore, the expression levels of p21 and MDM2, the protein products of p53 target genes, also increased with the increased expression of p53 after the treatment with CEP-1347 (Figure 3A,B). This increase in their expression levels was cancelled by the knockdown of p53, which was consistent with the idea that it reflected an enhancement in p53 transcriptional activity (Figure 3C). These results indicate that CEP-1347 activated the p53 pathway.

### 3.3. MDM4 Depletion Activates the p53 Pathway and Suppresses the Viability of UM Cells

**Figure 4:**
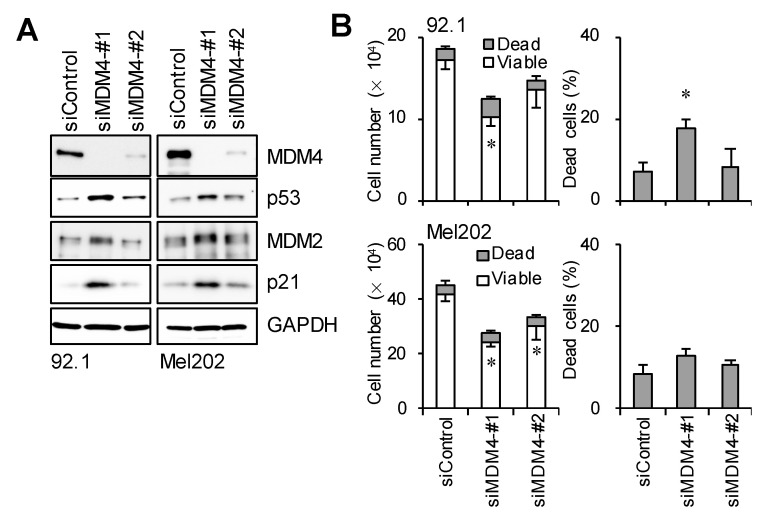
p53 activation by MDM4 depletion in uveal melanoma cells. (**A**) Immunoblot analysis of 92.1 and Mel202 cells transfected with an siRNA against MDM4 (#1 or #2) or a control siRNA (siControl) and cultured for 4 days. (**B**) Cells were transfected as in (**A**). Then, the numbers of viable and dead cells (left panels) as well as the percentage of dead cells (right panels) were determined by a trypan blue dye exclusion assay. Values represent the means ± SDs of triplicate samples from a representative experiment. * *p* < 0.05 vs. siControl by the Student’s *t*-test. Similar results were reproduced in two independent biological replicates.

We attempted to clarify whether the depletion of MDM4 induced by the CEP-1347 treatment directly activated the p53 pathway and suppressed the viability of UM cells. We transfected UM cells with siRNAs against MDM4 and examined the p53 pathway by immunoblotting. The results obtained demonstrated that the down-regulated expression of MDM4 was associated with the up-regulated expression of p53 and its target gene products, p21 and MDM2 (Figure 4A). To confirm the inhibitory effects of the knockdown of MDM4 on UM cell viability, we performed trypan blue staining and found that the knockdown of MDM4 reduced the number of viable cells and induced, though marginally, cell death in UM cells (Figure 4B). Thus, the results indicated that reduced MDM4 expression induced the upregulation of p53 expression and suppressed the viability of UM cells. However, compared to CEP-1347, the knockdown of MDM4 alone appeared to have weaker effects on p53 expression and the viability of UM, which led us to surmise that CEP-1347 may have another target(s) in addition to MDM4 to activate p53 and inhibit the viability of UM cells.

### 3.4. CEP-1347-Mediated Suppression of PKC Activity and MDM4 Expression Cooperatively Inhibits UM Cell Viability

**Figure 5:**
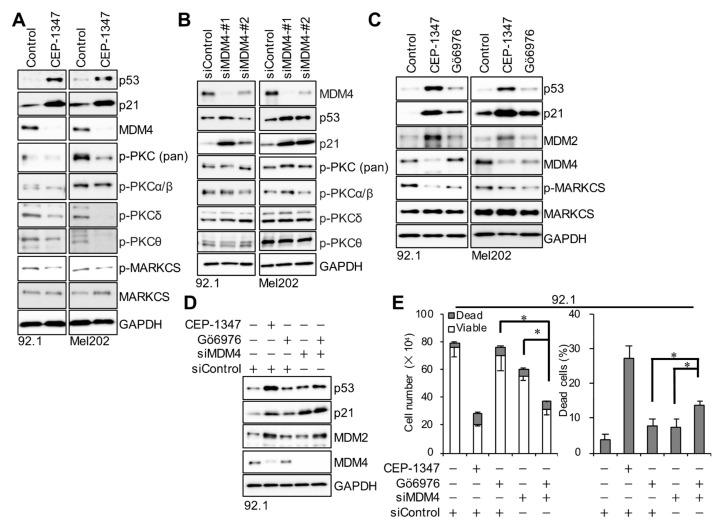
CEP-1347 inhibits PKC activity as well as MDM4 expression in uveal melanoma cells, which cooperate to activate p53 and inhibit cell viability. (**A**) Immunoblot analysis of 92.1 and Mel202 cells treated with CEP-1347 (0.5 μM, 3 days). (**B**) Immunoblot analysis of cells transfected with an siRNA against MDM4 (#1 or #2) or a control siRNA (siControl) for 4 days. (**C**) Immunoblot analysis of cells treated with CEP-1347 (0.5 μM) or Gö6976 (10 nM) for 3 days. (**D**) Immunoblot analysis of 92.1 cells transfected as indicated for 1 day and then treated with or without CEP-1347 (0.5 μM) or 10 nM Gö6976 (10 nM) for 3 days. (**E**) 92.1 cells were treated as in (**D**), and the trypan blue dye exclusion assay was conducted to calculate the numbers of viable and dead cells (left panels) as well as the percentage of dead cells (right panels). Values represent the means ± SDs of triplicate samples from a representative experiment. * *p* < 0.05 vs. siMDM4 + Gö6976 by the Student’s *t-*test. Similar results were reproduced in two independent biological replicates.

Somatic mutations in either of the two genes encoding the alpha subunit of the heterotrimeric G protein-coupled receptor (GPCR) complex, GNAQ or GNA11, have been detected in more than 90% of UM patients; furthermore, the PKC pathway downstream of these mutations was shown to be activated in UM and regulate cell growth [25,26,27,28,29]. Therefore, we investigated the involvement of the PKC pathway in the suppression of cell viability by CEP-1347. We initially examined the effects of CEP-1347 on the PKC pathway by immunoblotting and demonstrated that CEP-1347 reduced the phosphorylation of several PKCs, such as PKCδ and PKCθ, as well as that of MARCKS, one of the main substrates of PKCs (Figure 5A). In contrast, no changes were observed in the phosphorylation state of PKCs, even when p53 activation was observed by the knockdown of MDM4 (Figure 5B). This result suggests that the suppression of PKC activity by CEP-1347 in UM cells was independent of the reduced expression of MDM4. We then examined the mechanisms by which the inhibition of PKC by CEP-1347 affected its suppression of UM cell viability. To achieve this, we treated UM cells with Gö6976, a broad-spectrum PKC inhibitor, and examined its effects on cell viability when PKC activity was suppressed to the same extent as that by CEP-1347 (Figure 5C), as indicated by the expression of phosphorylated MARCKS. We demonstrated that this treatment had no apparent effect on the suppression of cell viability (compare column 1 vs. 3 in Figure 5E). We also observed that the knockdown of MDM4 had weaker effects on p53 expression and the viability of UM cells compared to CEP-1347 treatment (compare lane 2 vs. 4 in Figure 5D and column 2 vs. 4 in Figure 5E). We then inhibited PKC along with the knockdown of MDM4 and showed significantly stronger effects of this combination on the activation of the p53 pathway (compare lane 3 vs. 5 and lane 4 vs. 5 in Figure 5D), reduction in the number of viable cells, and induction of cell death (compare column 3 vs. 5 and column 4 vs. 5 in Figure 5E) than those of a treatment with each drug alone. Collectively, these results suggest that the concurrent knockdown of MDM4 expression and inhibition of PKC activity cooperated to induce p53 activation, reduce the number of viable cells, and induce cell death in UM cells. Notably, when the PKC inhibitor suppressed PKC activity to the same extent as CEP-1347, the levels of MDM4 suppression and p53 activation were not as significant (compare lane 1 vs. 3 in Figure 5C) as those by CEP-1347 (compare lane 2 vs. 3 in Figure 5C). These results indicate that the suppression of MDM4 expression by CEP-1347 was largely independent of PKC activity. Therefore, CEP-1347 appears to suppress MDM4 expression and PKC activity independently of each other, both of which are key to efficient activation of p53 and inhibition of cell viability in UM cells. Thus, the dual inhibitory activity of CEP-1347 towards MDM4 and PKC may contribute to the potent growth suppression of UM cells by CEP-1347.

## 4. Discussion

**Figure 6:**
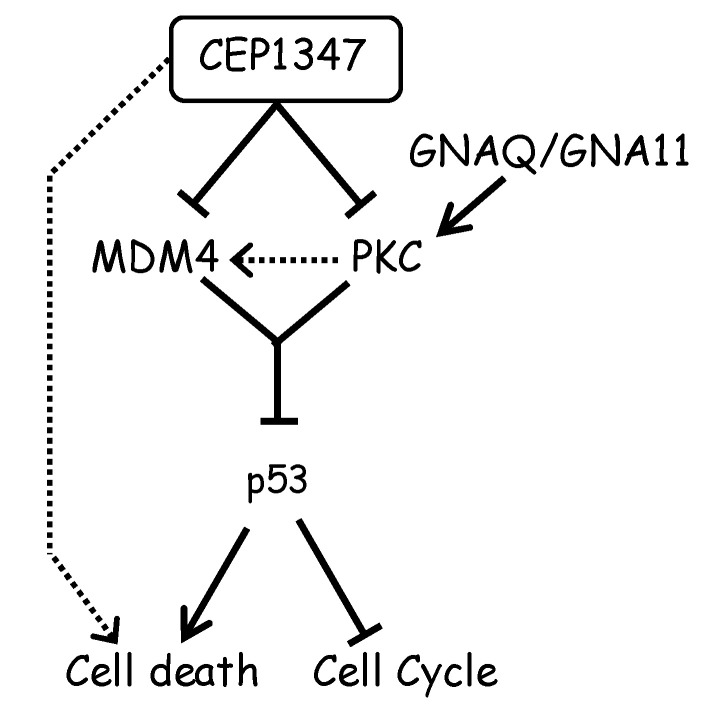
A proposed model for the mechanism of CEP-1347-mediated inhibition of uveal melanoma cell growth.

There are currently no effective treatments for advanced UM, including recurrent and metastatic UM, and novel therapeutic agents are urgently needed for eye preservation and the long-term prognosis of patients. Therefore, various agents, such as immune checkpoint inhibitors and those that target signaling pathways, have been developed. However, current therapies have limited efficacy. The development of new agents is required to address the unmet medical needs of advanced UM. In the present study, we focused on MDM4, which has been shown to play an important role in UM cell proliferation [11], and examined the effects of the MDM4 inhibitor CEP-1347 as a novel therapeutic strategy for malignant UM. We demonstrated that CEP-1347 exerted strong inhibitory effects on UM cell viability; however, the suppression of MDM4 function by knockdown alone did not exert the potent growth-inhibitory effects observed with CEP-1347. Therefore, we hypothesized that CEP-1347 exerted its inhibitory effects on UM cell viability not only by suppressing MDM4 expression but also through other mechanisms. Since the activation of PKC, a large group of serine/threonine kinases, occurs downstream of the GNAQ/GNA11 mutation found in the majority of UM [25,26,27,28], we focused on the Gα-PKC pathway. The results obtained demonstrated that (1) the treatment with CEP-1347 suppressed the activity of a wide range of PKCs, and (2) the suppression of PKC by CEP-1347 was independent of the suppression of MDM4 expression. Importantly, PKC suppression by the PKC inhibitor alone, as well as MDM4 expression suppression by knockdown alone, exerted weak inhibitory effects on UM cell viability, whereas their combination exerted strong inhibitory effects. We also showed that their combination activated the p53 pathway more efficiently than each treatment alone (Figure 5D). These results suggest that CEP-1347 suppresses MDM4 expression and PKC activity in UM cells, resulting in the combinatorial up-regulation of the p53 pathway and potent growth suppressive effects (Figure 6). Here, the mechanisms by which the suppression of MDM4 expression and PKC activity cooperatively increased p53 expression in UM cells remain unclear; however, since p53 protein is degraded through ubiquitination not only by MDM4/MDM2 heterodimers but also by MDM2 homodimers, PKC might possibly facilitate the formation of MDM2 homodimers and their subsequent degradation of p53 even when MDM4 expression is suppressed. In addition, since p53 is reportedly phosphorylated by PKC isoforms [30,31], the transcriptional activity of p53 could be modulated through PKC-mediated phosphorylation. Another question that remains unanswered is the role of mixed lineage kinase (MLK) in the cellular effects CEP-1347 causes on UM cells. CEP-1347 was originally developed as an MLK inhibitor [32,33], but the role of MLK in the observed effects of CEP-1347 on MDM4 expression and PKC activity remains to be elucidated. Furthermore, although the combination of MDM4 expression and PKC inhibition reduced the number of viable cells as strongly as CEP-1347, its effect on cell death was weaker than that of CEP-1347 (Figure 5E). These results suggest that CEP-1347 may have other targets in addition to MDM4 expression and PKC activity (Figure 6).

We herein demonstrated for the first time that CEP-1347 potently activated p53 and suppressed PKC activity in UM cells with GNAQ mutations 92.1 and Mel202 [28,34]. More than 90% of UM tumors have mutations in one of the two genes encoding the alpha subunit of the heterotrimeric GPCR complex, GNAQ or GNA11. This mutation most likely occurs early in the formation of UM; however, its association with UM metastasis and survival remains controversial [35]. Nevertheless, these mutations anchor Gα to the active form, thereby activating various signals, such as the PKC pathway [25,26,27,28]. The PKC and MEK pathways were found to be activated in UM with Gα mutations, which was sensitive to inhibitors of these pathways [28]. However, the efficacy of newly developed PKC inhibitors, such as AEB071 and IDE196, has been limited in clinical trials [36,37]. Furthermore, clinical trials on the combination of PKC and MEK inhibitors were discontinued early due to strong toxicity (NCT01801358) [36,38]. On the other hand, the efficacy of the combination of NVP-CGM097, which activates p53 by binding to MDM2, and PKC inhibitors was reported to be effective in preclinical models [39]. However, the molecular mechanisms of action of agents that target the MDM2-p53 interaction inevitably induce the activation of p53 in normal tissues, resulting in severe adverse effects; therefore, inhibitors that are both safe and effective have not yet been developed [40,41]. Since MDM4, which assists the function of MDM2, is up-regulated in UM [11], Heijkants et al. proposed a therapeutic strategy to control UM by targeting MDM4 and simultaneously inhibiting PKC. However, since there were no safe MDM4 inhibitors available at the time, the combination of Nutlin3a, a classical MDM2-p53 inhibitor, and AEB071, a broad-spectrum PKC inhibitor, was used [42]. Only a limited number of MDM4 inhibitors are currently available, and to the best of our knowledge, there is no MDM4 inhibitor with safety information in humans other than CEP-1347. We propose the potential of CEP-1347 as an effective option for UM; it is a novel MDM4 inhibitor that has been shown to down-regulate MDM4 and activate p53 in retinoblastoma, glioblastoma, and malignant meningioma cells [16,17,18]. Regarding the in vivo efficacy of CEP-1347, our recent study successfully demonstrated the antitumor effects of CEP-1347 in a mouse xenograft model of meningioma. Therefore, we anticipate similar therapeutic effects in vivo for other cancer types. We herein demonstrated that CEP-1347 suppressed not only MDM4 expression but also PKC activity within the clinically relevant concentration range and thus represents one of the most potentially effective agents for high-grade UM. Since CEP-1347 has a well-characterized safety profile in humans demonstrated in a phase 2 clinical trial for the treatment of Parkinson’s disease [19], it may immediately advance to the bedside. Therefore, CEP-1347 has potential against advanced and metastatic UM, for which there are currently no effective treatments available.

p53 mutations are very rare, and MDM4 is overexpressed in UM [6,11]. Therefore, we hypothesized that, similar to its effects in retinoblastoma, CEP-1347 may also effectively suppress MDM4, which is a functional p53 inhibitor. The present results show that CEP-1347 up-regulated p53 expression by suppressing both MDM4 expression and PKC activity in UM cells, thereby potently inhibiting cell viability. However, since p53 activation by CEP-1347 increased the expression of its target gene *MDM2*, the major negative regulator of p53 (Figure 3A,B), the activation of p53 may be affected by negative feedback via MDM2. Nevertheless, the results of the colony formation assay showed that the transient treatment with CEP-1347 effectively suppressed the clonogenicity of UM cells for a prolonged period of time (Figure 2D). Therefore, the up-regulation of MDM2 by CEP-1347 may not necessarily shorten the duration of intracellular p53 activity by inducing p53 degradation in UM cells. Instead, it may reduce the level of intracellular p53 activity to such an extent that the induction of cell death but not cell cycle arrest is prevented. To suppress the negative feedback of p53 via MDM2 in the hope of boosting the growth-inhibitory activity of p53, we combined CEP-1347 with an MDM2 inhibitor and investigated the effects of the combination on meningioma and glioblastoma cells [43]. The results demonstrated that the combination of CEP-1347 and RG7112, an MDM2-p53 inhibitor, significantly activated the p53 pathway and suppressed cell viability despite the up-regulated expression of MDM2 via the p53 pathway. Although p53-reactivating small molecules that target MDM2, such as Nutlin3a, have been examined in clinical trials, many exert strong adverse effects, including myelosuppression [13,14,15]. Recent studies have focused on the development and clinical testing of novel p53-activating small molecules for AML, multiple myeloma, and other hematological malignancies [44]. Alrizomadlin (APG-115), a small-molecule inhibitor that selectively targets the p53-MDM2 interaction, has been approved by the US FDA and exhibited preliminary antitumor activity in UM [6]. Additional information on the safety and antitumor effects of these MDM2 inhibitors in UM patients may facilitate the translation of combinations of CEP-1347 and MDM2 inhibitors into the treatment of UM.

## 5. Conclusions

In the present study, we demonstrated for the first time that CEP-1347 suppressed MDM4 expression and PKC activity, thereby activating the p53 pathway in p53 wild-type UM cells. This ultimately reduced cell proliferation and survival. The present results provide supportive evidence for the use of CEP-1347 as a novel therapeutic option for UM because many UM cases are characterized by the functional suppression of p53 due to the overexpression of MDM4 and also by the activation of the PKC pathway due to GNAQ/GNA11 mutations.

## Data Availability

Data are contained within the article.

## Supplementary Materials

The following supporting information can be downloaded at: https://www.mdpi.com/article/10.3390/cancers16010118/s1, Figure S1: Original Western Blot images.
