# Supplementary material for: CEP-1347 Dually Targets MDM4 and PKC to Activate p53 and Inhibit the Growth of Uveal Melanoma Cells
Source: Cancers (Basel). 2023 Dec 25;16(1):118. doi: 10.3390/cancers16010118 (PMC10778035; doi:10.3390/cancers16010118)
Supplement: Supplementary file 1 [file cancers-16-00118-s001.zip › cancers-2741883-supplementary.pdf]

Original blots

Figure 1

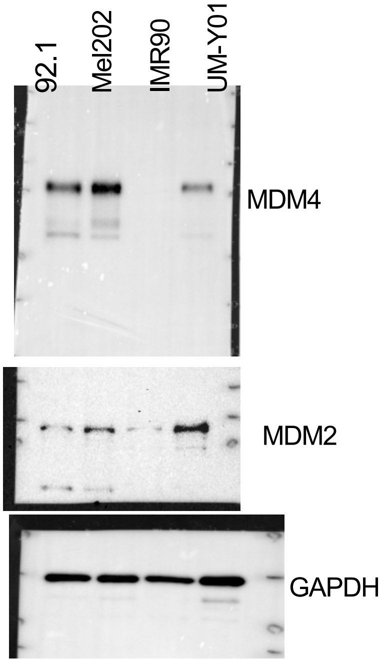

Figure 3A

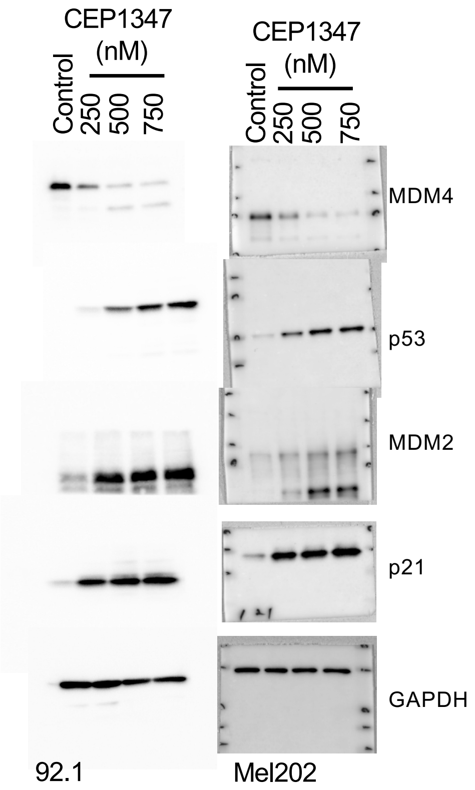

Figure 3B

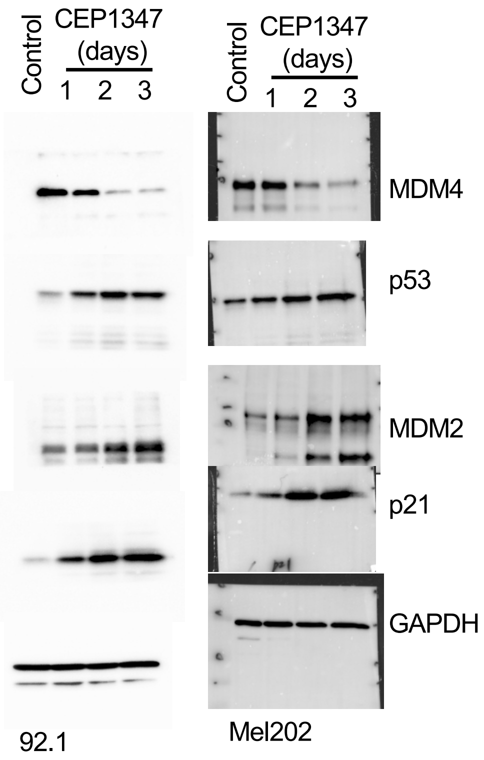

Figure 3C

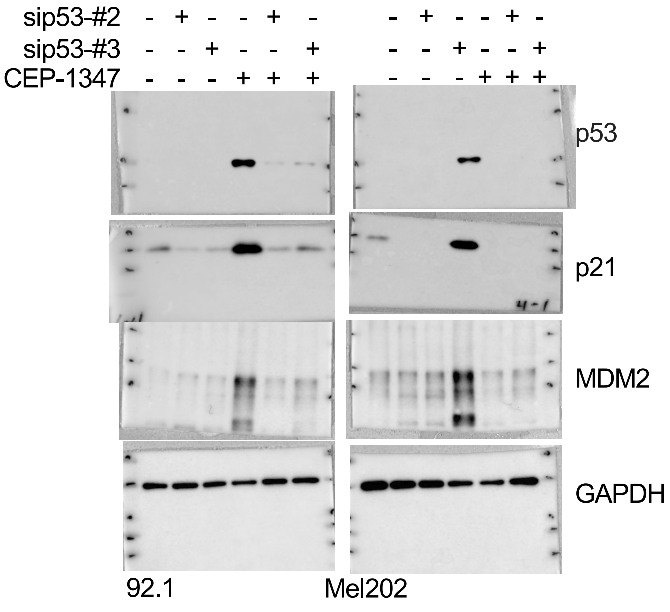

Figure 4A

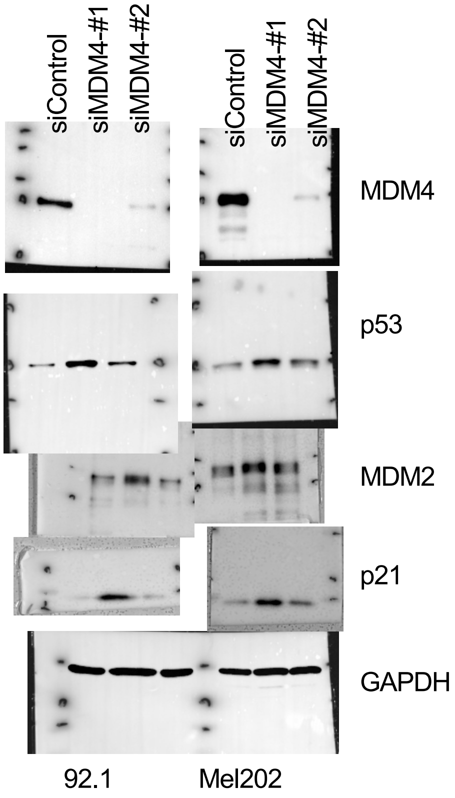

Original blots, contineued

Figure 5A

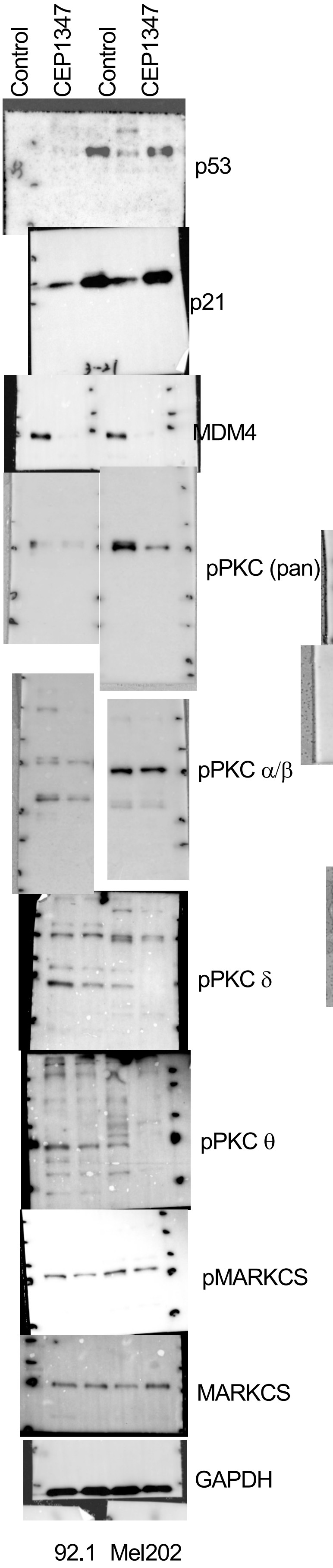

Figure 5B

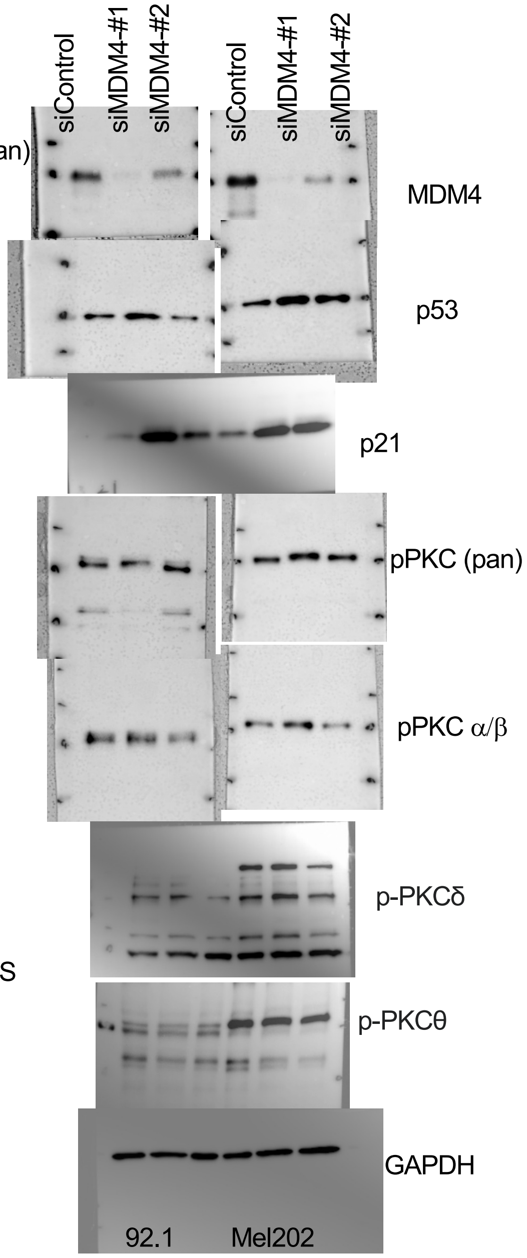

Figure 5C

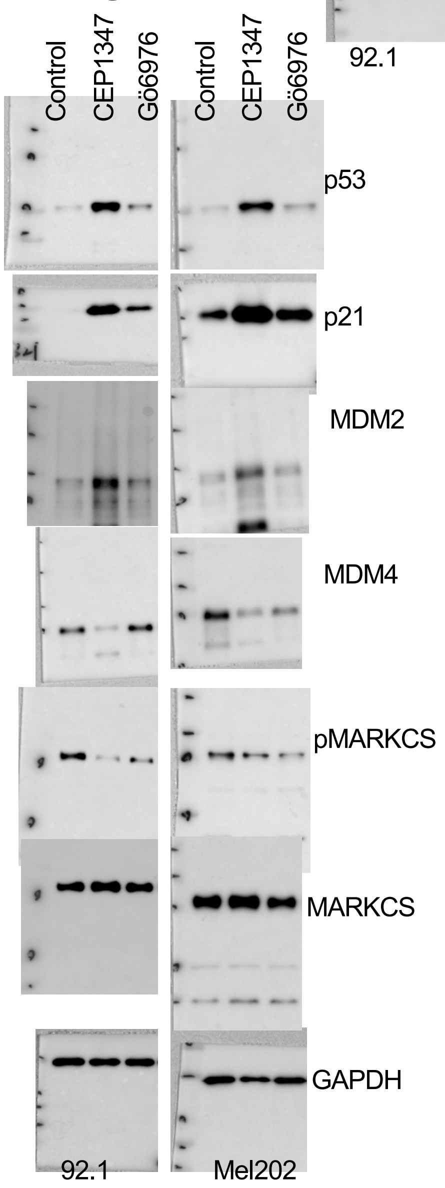

Figure 5D

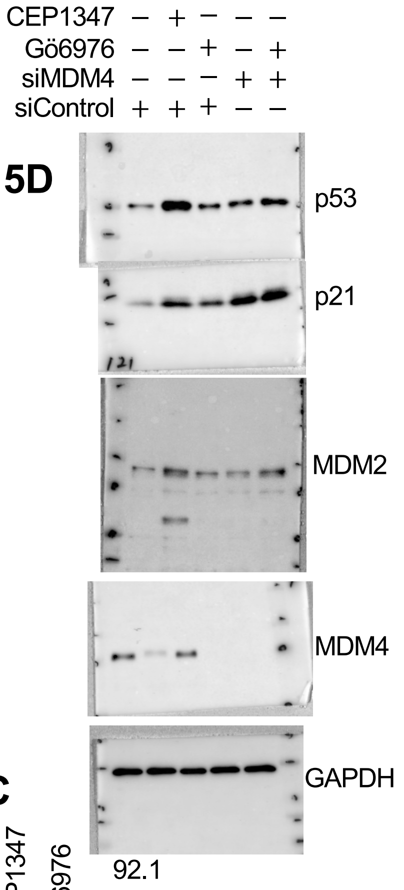

Figure S1: Original Western Blot images.
